# Complete Genome Sequences of Streptococcus pneumoniae Strains HU-OH (Serotype 3, Sequence Type 183 [ST183]), NU83127 (Serotype 4, ST246), and ATCC 49619 (Serotype 19F, ST1203)

**DOI:** 10.1128/MRA.01504-18

**Published:** 2019-02-28

**Authors:** Kentaro Nagaoka, Satoshi Konno, Kazunori Murase, Taisei Kikuchi, Yoshitomo Morinaga, Katsunori Yanagihara, Ichiro Nakagawa

**Affiliations:** a Department of Respiratory Medicine, Faculty of Medicine and Graduate School of Medicine, Hokkaido University, Sapporo, Hokkaido, Japan; b Department of Infectious Diseases, Faculty of Medicine, University of Miyazaki, Miyazaki, Japan; c Department of Laboratory Medicine, Nagasaki University Graduate School of Biomedical Sciences, Nagasaki, Japan; d Department of Microbiology, Graduate School of Medicine, Kyoto University, Kyoto, Japan; University of Delaware

## Abstract

Streptococcus pneumoniae is a pathogenic bacterium frequently found in the respiratory tract of humans and commonly causes pneumonia and bacterial meningitis. Here, the complete circular genome sequences of three S. pneumoniae strains with different serotypes and sequence types have been reported.

## ANNOUNCEMENT

Streptococcus pneumoniae is a Gram-positive bacterium that is responsible for the majority of community-acquired pneumonia in elderly people and acute otitis media in children and causes invasive infections, such as meningitis and sepsis (1, 2). Appropriate and immediate antibiotic intervention is required to treat infection by invasive pathogens with multidrug resistance (3, 4). Thus, the administration of multivalent vaccines has been recommended for the prevention of pneumococcal infection. Although a 23-valent pneumococcal polysaccharide vaccine (PPV23) can reduce the risk of invasive pneumococcal diseases among adults, its effectiveness is still controversial (5). Additionally, the recently approved pneumococcal conjugate vaccine (PCV13) is expected to prevent approximately half of the pneumococcal pneumonia cases in the elderly (6, 7). In Japan, capsular serotypes 3 and 19F are the most and the third most common serotypes, respectively, isolated from adult patients with community-acquired pneumonia (8). However, rare serotypes, such as serotype 4, were identified as pneumonia pathogens, even after PCV13 or PPV23 administration. Here, the complete circular genome sequences of S. pneumoniae strains HU-OH (serotype 3 and sequence type 183 [ST183]), NU83127 (serotype 4 and ST246), and ATCC 49619 (serotype 19F and ST1203) have been reported. Characteristics of these strains are shown in Table 1.

**TABLE 1:** Relevant characteristics of three S. pneumoniae strains

Characteristic^*a*^	Data for strain:		
	NU83127	HU-OH	ATCC 49619
Strain information			
Serotype	4	3	19F
Sequence type	246	183	1203
Origin	Sputum	Sputum	Sputum
Clinical symptom	Severe pneumonia	Severe pneumonia	ND^*b*^
General genome and assembly statistics			
Genome size (bp)	2,180,701	2,058,492	2,096,333
No. of reads			
MinION	103,064	44,589	108,555
MiSeq	1,992,709	1,946,015	1,969,090
Total no. of bases			
MinION	608,074,243	192,193,468	453,807,381
MiSeq	1,199,610,818	1,171,501,030	1,185,392,180
Coverage (×)	828.94	662.47	781.93
G+C content (%)	39.7	39.72	39.67
No. of CDSs	2,141	2,025	2,068
No. of rRNAs	12	12	12
No. of tRNAs	59	58	59
No. of tmRNAs	1	1	1
Accession no.	AP018936	AP018937	AP018938

a CDSs, coding sequences; tmRNA, transfer-messenger RNA.

b ND, not determined.

In Japan, two S. pneumoniae strains, namely, HU-OH and NU83127, were isolated from the sputum of patients with severe pneumonia in 2014 and 1997, respectively. A sheep blood agar plate (Nissui) was used for their isolation. S. pneumoniae strain ATCC 49619 was obtained from the American Type Culture Collection. These strains were incubated for 8 hours at 37°C in Todd-Hewitt broth with 0.2% yeast extract (Becton, Dickinson and Company). Genomic DNA was extracted using a DNeasy tissue and blood kit according to the manufacturer’s instructions (Qiagen). Short and long read sequences were generated on MiSeq (Illumina) and MinION (Oxford Nanopore Technologies) platforms, respectively. The Illumina library was prepared using the Nextera DNA library kit, and MiSeq paired-end reads were generated using the MiSeq reagent kit (v3, 600 cycles). A MinION library was prepared from unsheared genomic DNA using the rapid barcoding kit (SQK-RBK001) and sequenced with an R9 flow cell (FLO-MIN106). The Illumina data were preprocessed using Trimmomatic (v0.36) to remove adapter and low-quality sequences (9) and hybrid assembled with MinION long reads using the Unicycler pipeline (v0.4.7b) with default parameters (10). Next, the assembled genome sequence was annotated using Prokka (v1.11) with default settings (11). Assembly statistics and general genome information are listed in Table 1.

In 2014 in Japan, vaccinations with PCV13 were initiated for elders over 65 years old. S. pneumoniae strains HU-OH and NU83127 were isolated shortly before the administration of PCV13; therefore, the complete genome sequences of these strains will provide useful information for future analysis of genetic changes in S. pneumoniae.

### Data availability.

The complete circular genome sequences of S. pneumoniae strains NU83127, HU-OH, and ATCC 49619 have been deposited in DDBJ/EMBL/GenBank under the accession numbers AP018936, AP018937, and AP018938, respectively. The raw Illumina and MinION read data can be found in the DDBJ Sequence Read Archive with the accession number DRA007465.

## References

[1] Centers for Disease Control and Prevention. 2015 Kroger A, Hamborsky J, Wolfe C (ed). Epidemiology and Prevention of Vaccine-Preventable Diseases, 13th ed Public Health Foundation, Washington, DC.

[2] Lynch JP 3rd, Zhanel GG 2009 *Streptococcus pneumoniae*: epidemiology, risk factors, and strategies for prevention. Semin Respir Crit Care Med 30:189–209. doi:10.1055/s-0029-1202938. 19296419

[3] Moroney JF, Fiore AE, Harrison LH, Patterson JE, Farley MM, Jorgensen JH, Phelan M, Facklam RR, Cetron MS, Breiman RF, Kolczak M, Schuchat A 2001 Clinical outcomes of bacteremic pneumococcal pneumonia in the era of antibiotic resistance. Clin Infect Dis 33:797–805. doi:10.1086/322623. 11512085

[4] Cillóniz C, Ardanuy C, Vila J, Torres A 2016 What is the clinical relevance of drug-resistant pneumococcus? Curr Opin Pulm Med 22:227–234. doi:10.1097/MCP.0000000000000262. 26901109

[5] Moberley S, Holden J, Tatham DP, Andrews RM 2013 Vaccines for preventing pneumococcal infection in adults. Cochrane Database Syst Rev 1:CD000422. doi:10.1002/14651858.CD000422.pub3. PMC7045867 23440780

[6] Weinberger DM, Shapiro ED 2014 Pneumococcal conjugate vaccines for adults: reasons for optimism and for caution. Hum Vaccin Immunother 10:1334–1336. doi:10.4161/hv.28962. 24763136 PMC4896592

[7] Hak E, Grobbee DE, Sanders EAM, Verheij TJM, Bolkenbaas M, Huijts SM, Gruber WC, Tansey S, McDonough A, Thoma B, Patterson S, van Alphen AJ, Bonten MJM 2008 Rationale and design of CAPITA: a RCT of 13-valent conjugated pneumococcal vaccine efficacy among older adults. Neth J Med 66:378–383. http://www.njmonline.nl/article.php?a=713&d=442&i=106. 18990781

[8] Morimoto K, Suzuki M, Ishifuji T, Yaegashi M, Asoh N, Hamashige N, Abe M, Aoshima M, Ariyoshi K; Adult Pneumonia Study Group–Japan. 2015 The burden and etiology of community-onset pneumonia in the aging Japanese population: a multicenter prospective study. PLoS One 10:e0122247. doi:10.1371/journal.pone.0122247. 25822890 PMC4378946

[9] Bolger AM, Lohse M, Usadel B 2014 Trimmomatic: a flexible trimmer for Illumina sequence data. Bioinformatics 30:2114–2120. doi:10.1093/bioinformatics/btu170. 24695404 PMC4103590

[10] Wick RR, Judd LM, Gorrie CL, Holt KE 2017 Unicycler: resolving bacterial genome assemblies from short and long sequencing reads. PLoS Comput Biol 13:e1005595. doi:10.1371/journal.pcbi.1005595. 28594827 PMC5481147

[11] Seemann T 2014 Prokka: rapid prokaryotic genome annotation. Bioinformatics 30:2068–2069. doi:10.1093/bioinformatics/btu153. 24642063

